# Trends of Colistin MIC Among *Acinetobacter Baumannii* and *Pseudomonas aeruginosa* at a First-Class Hospital in Vietnam

**DOI:** 10.1155/jotm/6165665

**Published:** 2025-03-16

**Authors:** Tuan Huu Ngoc Nguyen, Huy Quang Nguyen, Ngan Thi Thu Le, Han To Ngoc Nguyen, Hung Cao Dinh, Tam Ngoc Nguyen, Ha Minh Nguyen

**Affiliations:** ^1^Biomedical Research Center, Pham Ngoc Thach University of Medicine, Ho Chi Minh City, Vietnam; ^2^Laboratory Department, Nguyen Tri Phuong Hospital, Ho Chi Minh City, Vietnam; ^3^Department of Internal Medicine, Faculty of Medicine, Pham Ngoc Thach University of Medicine, Ho Chi Minh City, Vietnam; ^4^Department of Internal Medicine, Faculty of Medicine, Nguyen Tat Thanh University, Ho Chi Minh City, Vietnam

**Keywords:** *A. baumannii*, colistin, minimum inhibitory concentration (MIC), *P. aeruginosa*

## Abstract

**Introduction:** *A. baumannii* and *P. aeruginosa* belong to the multidrug-resistant Gram-negative bacteria group, posing significant challenges in treatment. Colistin is considered the last-line antibiotic for treating this bacterium. It is essential to determine the minimum inhibitory concentration (MIC) to adjust the appropriate dosage.

**Method:** A cross-sectional descriptive study using data from January 2020 to December 2024 was conducted.

**Results:** The infections caused by *A. baumannii* and *P. aeruginosa* showed an increasing trend over the years, accounting for 17.4% and 9.6% of common multidrug-resistant Gram-negative bacteria, respectively. *A. baumannii* exhibited higher resistance rates than *P. aeruginosa* with multiple tested antibiotics. Although no Colistin-resistant strains were observed for either bacterium of interest during the observation period, both bacteria of interest showed a statistically significant change during the survey period (*p* < 0.05). In addition, the MIC value of ≤ 0.75 μg/mL was the most prevalent over 80% from 2020 to 2021, but its percentage declined strongly by 60%–65% in the next 3 years (*p* < 0.0001). Meanwhile, the MIC value of 1.0 μg/mL became the most common over 70% with a statistically significant difference (*p* < 0.0001). Regarding the MIC value based on infection types, the MIC value for *P. aeruginosa* causing septicemia was considerably concentrated at 1.0 μg/mL at 84.6%, while its percentage was lower in *A. baumannii* at 37.9% (*p* < 0.0001). Looking into MIC values based on carbapenem-resistant proportions, the MIC values from 1.0 to 2.0 μg/mL were higher in imipenem-resistant strains of both bacteria of interest compared with nonresistant strains (*p* < 0.0001). This difference was also observed in meropenem-resistant *A. baumannii* but was not demonstrated in *P. aeruginosa*.

**Conclusions:** Although no colistin-resistant strains were observed, *A. baumannii* and *P. aeruginosa* showed statistically significant changes in the most prevalent colistin MIC values, which have been approaching the resistance threshold over the years. It is essential to implement control measures of colistin usage before bacteria become completely resistant.

## 1. Introduction

Antibiotic resistance is currently one of the most globally concerning health issues. Jim O'Neill's comprehensive report on the worldwide antibiotic resistance situation estimates that by 2050, there will be over 10 million deaths globally due to drug-resistant bacteria, resulting in damages exceeding 100 trillion dollars in treatment costs. In this context, Asia is considered the region with the highest rates of antibiotic-resistant bacteria, especially in developing countries like Vietnam [1]. Reports on the antibiotic resistance situation in Vietnam have documented the widespread prevalence of Gram-negative bacteria in the majority of healthcare facilities, showing high resistance to commonly used antibiotics [2].

Among the common Gram-negative bacteria, the most prominent are *Acinetobacter baumannii* and *Pseudomonas aeruginosa*. Both are listed by the World Health Organization (WHO) as multidrug-resistant bacteria requiring top priority in research and development of new antibiotic drugs. These bacteria are known to be among the superbugs causing multidrug-resistant diseases, exhibiting broad resistance to various commonly used antibiotics such as β-Lactams, aminoglycosides, and fluoroquinolones. They also possess a high survival capability under various environmental conditions [3, 4]. These factors have posed considerable challenges in the treatment of infections caused by *A. baumannii* and *P. aeruginosa*. In this situation, colistin is considered one of the last-line antibiotics for treating multidrug-resistant Gram-negative strains [5]. Besides its pharmacological effects, colistin also exhibits certain side effects on the human body, particularly nephrotoxicity [6]. Consequently, administering Colistin to patients requires maintaining drug concentrations through the assessment of minimum inhibitory concentration (MIC) to adjust appropriate doses for effective bacterial eradication while minimizing drug toxicity. In recent years, cases of Gram-negative bacillus being clinically susceptible to colistin have been documented in the medical literature [7, 8]. Based on many complex bacterial antibiotic resistance mechanisms, plus inappropriate antibiotic use in some places, colistin's MIC levels have shifted in an upward direction over time. Mild to severe colistin resistance has been reported to be associated with expression of mcr-family genes, especially mcr-1 [9]. Therefore, in addition to helping to limit the toxicity of the drug, periodic monitoring of the MIC of this antibiotic in healthcare facilities also helps to monitor the trend and detect colistin resistance early.

Currently, reports on the antibiotic resistance situation in Vietnam primarily focus on the sensitivity and resistance rates of *A. baumannii* and *P. aeruginosa*, with limited attention given to the variations in colistin MIC values over the years. This emphasizes the need for research into the changes in colistin MIC values for these two bacterial species. Such research is crucial for implementing effective antibiotic management strategies to prevent the escalation of resistance. This study aims to determine the trends in common MIC values of Colistin for *A. baumannii* and *P. aeruginosa* in clinical specimens from 2020 to 2024.

## 2. Methodology

### 2.1. Ethical Approval

The research was accepted by the ethical principles established by the Ethics Committee for Biomedical Research at Nguyen Tri Phuong Hospital, as indicated by document number 746/NTP-HĐĐĐ, dated April 25, 2023.

### 2.2. Study Design

This cross-sectional descriptive study used recorded data from January 2020 to December 2024.

### 2.3. Bacterial Strains

Antibiotic susceptibility testing data were analyzed for 2027 nonrepeated strains of *A. baumannii* and 946 nonrepeated strains of *P. aeruginosa*, which were isolated from various clinical samples collected from patients treated at Nguyen Tri Phuong Hospital, Ho Chi Minh City, Vietnam, between January 2020 and December 2024. The bacterial strains were identified and subjected to antibiotic susceptibility testing following the standard operating procedures of the hospital's Laboratory Department. The stored samples were not used in this study, and each sample was tested only once. All samples provide comprehensive information including clinical department, cultured specimen type, bacterial identification results, and antibiotic susceptibility profile. In addition, MIC testing with colistin was performed.

### 2.4. Bacterial Cultivation and Isolation

The clinical specimens were cultured on suitable nutrient media, including Blood Agar (BA), Chocolate Agar (CA), and Mac Conkey Agar (MC), using various cultivation techniques. For sputum and urine specimens, quantitative cultures were conducted on nutrient media, and the bacterial colonies were isolated for analysis when the colony count exceeded 10^4^ CFU/mL. For other specimen types, both isolation and analysis of all bacterial colonies were performed.

### 2.5. Bacterial Identification

The selected bacterial colonies underwent Gram staining. Based on the Gram staining results and the type of growth medium, Gram-negative bacterial colonies were selected for identification using the IDS GN15 kit (Nam Khoa Biotek, Vietnam). There are 14 biochemical tests, including oxidase test, glucose fermentation test, nitrate reduction test, ONPG test, urease test, PAD test, citrate utilization test, Esculin hydrolysis test, H_2_S production test, indole production test, Voges–Proskauer test, malonate utilization test, lysin decarboxylase test, and motility test. Bacterial samples were incubated at 37°C for 18–20 h, and the results of the tested biochemical reactions were used to determine the scientific names of the bacteria.

### 2.6. Antibiotic Susceptibility Testing

To determine bacterial sensitivity to antibiotics, the disk diffusion method was employed by the Kirby–Bauer technique on commercial cation-adjusted Mueller–Hinton agar (MHA) provided by Nam Khoa Biotek, Vietnam. The selected bacterial colonies were diluted with physiological saline to create an inoculum with 0.5 McFarland. The suspension was spread onto MHA plates, and antibiotic disks (Nam Khoa Biotek, Vietnam) were placed on the agar. Antibiotic disks were selected based on clinical needs and the suspected bacterial species as follows.

For bacteria suspected to belong to the *Pseudomonas* spp genus (with positive oxidase reaction), place antibiotic disks including piperacillin/tazobactam, amoxicillin/clavulanate, ticarcillin/clavulanate, trimethoprim/sulfamethoxazole, amikacin, gentamicin, tobramycin, netilmicin, ciprofloxacin, levofloxacin, ceftazidime, cefepime, imipenem, meropenem, and colistin.

For bacteria suspected to belong to the *Acinetobacter* spp genus (with negative oxidase reaction), place antibiotic disks including piperacillin/tazobactam, trimethoprim/sulfamethoxazole, doxycycline, gentamicin, tobramycin, ciprofloxacin, levofloxacin, cefotaxime, ceftazidime, cefepime, imipenem, meropenem, and colistin.

Incubate the MHA plates at 37°C for a duration of 18–20 h. Compare the measured zone diameters with the interpretive criteria outlined in the Clinical and Laboratory Standards Institute (CLSI) 2024 guidelines [10]. *P. aeruginosa* ATCC 27853, *A. baumannii* ATCC 19606, and *Escherichia coli* ATCC 25922 were used as control strains according to the procedures of the diagnostic laboratory.

### 2.7. MIC of Colistin (Colistin MIC)

The MIC of colistin was conducted by E-Test method (Epsilometer test Epsilon) with reagents supplied by Nam Khoa Biotek, Vietnam. This method operates on the principle of using strips with a gradually decreasing antibiotic concentration along their length. A plastic strip was placed on an agar plate inoculated with the test organism and the antimicrobial diffuses into the agar in a concentration gradient along the strip. After incubation, an elliptical zone of inhibition forms around the strip, so the MIC is determined by reading the point where the zone of inhibition intersects the scale printed on the strip. The strip had a numerical scale of concentrations directly marked on it to assist in determining the MIC. The MHA plates were incubated at 37°C for 18–20 h, and the MIC values of colistin were read. The results were interpreted following the CLSI 2024 guidelines [10].

### 2.8. Statistical Analysis

The data was analyzed by STATA 14.2. Variables in the study included infection type, specimen type, antibiotic susceptibility phenotypes, MIC values over time, and carbapenem-resistant phenotypes. Descriptive statistical methods were employed to analyze each variable. The analysis results were presented in tables and bar charts depicting the positive rates by bacterial species, positive rates by specimen type, the distribution of colistin MIC values (MIC ≤ 0.75 μg/mL, MIC = 1.0 μg/mL, MIC = 1.5 μg/mL, and MIC = 2.0 μg/mL) from 2020 to 2024 and the rates of susceptibility (S: susceptible), intermediate (I: intermediate), and resistance (R: resistance) to the tested antibiotics. The Chi-square test and Fisher's exact test were employed to compare the differences in MIC value over time, as well as the difference between percentage of colistin MIC values and carbapenem-resistant strains. *p* value < 0.05 was considered a statistically significant difference.

## 3. Results

### 3.1. Percentage of Positive Sample

**Figure 1:**
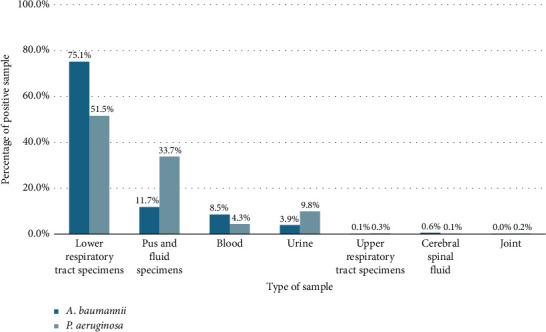
The positive culture rate of *A. baumannii* and *P. aeruginosa* by type of clinical specimen. The lower respiratory tract specimen includes sputum, bronchoalveolar lavage fluid, and endotracheal aspirate. The pus and fluid specimen group includes all types of pus, pleural fluid, peritoneal fluid, pericardial fluid, ascitic fluid, and catheter tip. The upper respiratory tract specimen consists of throat swabs and nasal discharge.

**Figure 2:**
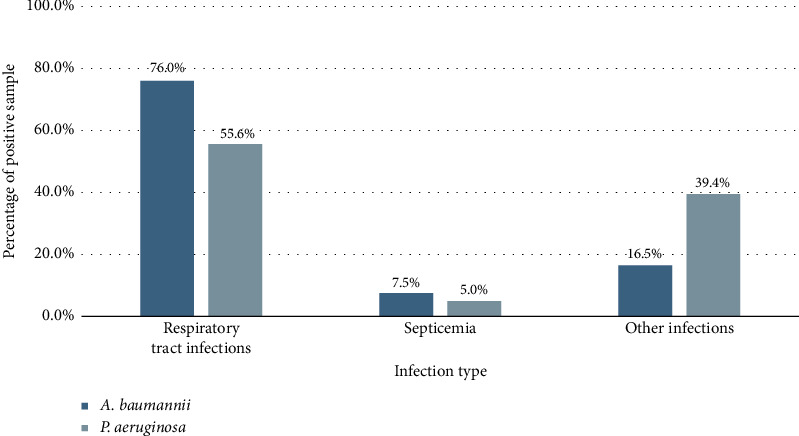
Distribution of positive culture rates of *A. baumannii* and *P. aeruginosa* across different infection types.

From 2020 to 2024, infections caused by *A. baumannii* have increased over time while those caused by *P. aeruginosa* remained stable. Respiratory infections were the most common type of disease. The proportion of these bacteria among the total common multidrug-resistant Gram-negative bacteria showed a gradual increase, with a 5-year average of 17.4% for *A. baumannii* and 9.6% for *P. aeruginosa*. In addition, the majority of cases were monoinfections, among which *A. baumannii* accounted for 71.9% and *P. aeruginosa* accounted for 70.3%. Coinfections, involving the bacteria of interest and other Gram-negative or Gram-positive bacteria, accounted for less than 30% of cases (Table 1). Among the sample types, lower respiratory specimens constituted the highest percentage, at 75.1% for *A. baumannii* and 51.5% for *P. aeruginosa*. The pus and fluid specimens accounted for 11.7% and 33.7% for *A. baumannii* and *P. aeruginosa*, respectively. Other samples, such as blood and urine, each contributed less than 10%, while specimens from the upper respiratory tract, CSU, and joints represented less than 1% (Figure 1). Regarding infection type, *A. baumannii* was a significant cause of respiratory tract infections, responsible for 76.0%, which was higher than the 55.6% attributed to *P. aeruginosa*. The percentage of septicemia cases was below 10% for both bacteria (Figure 2).

### 3.2. Antibiotic Susceptibility

In general, *A. baumannii* showed higher resistance to the tested antibiotics than *P. aeruginosa*. However, no colistin-resistant strains were observed in either of the bacteria of interest during the observation period (Table 2). Specifically, *A. baumannii* showed resistance rates over 80% to representatives of the fluoroquinolone group (ciprofloxacin and levofloxacin) and the β-lactam group (cefotaxime, ceftazidime, cefepime, imipenem, and meropenem). Notably, 43.4% of *A. baumannii* strains were resistant to doxycycline, which had the lowest resistance rate among the tested agents. *P. aeruginosa* demonstrated resistance rates below 50% to most tested antibiotics except for amoxicillin/clavulanate and trimethoprim/sulfamethoxazole.

### 3.3. MIC of Colistin

**Figure 3:**
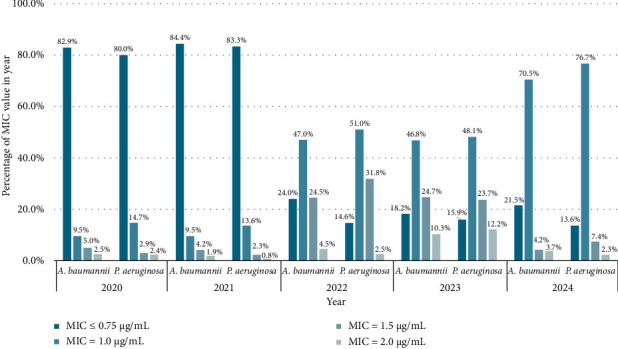
The trend of colistin MIC values of *A. baumannii* and *P. aeruginosa* from January 2020 to December 2024.

There was a significant change in the distribution of MIC value over the 5-year period for the two bacteria of interest. Overall, an MIC value of ≤ 0.75 μg/mL was the most prevalent trend during the first 2 years. In particular, its percentage significantly declined, and an MIC value of 1 μg/mL became the most common in the subsequent 3 years (Figure 3). The MIC distribution of colistin for the two bacteria of interest did not show a statistically significant difference between 2020 and 2021. However, a statistically significant decrease (*p* < 0.0001) was observed in the following years, with *p* value < 0.05 from 2021 to 2024 (Table 3).

During the survey period, *A. baumannii* showed a significant change in colistin MIC values, with the most prevalent MIC value increasing from ≤ 0.75 μg/mL to 1.0 μg/mL. In the first year, colistin MIC values were predominantly ≤ 0.75 μg/mL, accounting for 82.9%, while the proportions of other MIC values remained below 10% for each category. This distribution presented no statistically significant differences in 2021 (*p*=0.909). In 2022, the MIC value of ≤ 0.75 μg/mL dropped sharply by 60.8%, while the MIC values of 1.0 μg/mL and 1.5 μg/mL increased significantly at 47.0% and 24.5%, respectively (*p* < 0.0001). In 2023, there was a continued decline in the MIC value of ≤ 0.75 μg/mL by 5.8%, alongside an increase in other values, particularly a 5.8% rise in the MIC value of 2.0 μg/mL (*p*=0.004). However, in 2024, Colistin MIC values recovered to lower values. This was marked by a dramatic increase in the percentage of MIC = 1.0 μg/mL, from 46.8% to 70.5%, and a slight rise in the MIC value of ≤ 0.75 μg/mL by 3.3% (*p* < 0.0001). Meanwhile, the MIC values of 1.5 μg/mL and 2.0 μg/mL declined to below 10% in each category (Figure 3 and Table 3).

*P. aeruginosa* performed a similar trend to *A. baumannii* over a 5-year period. In 2020, the most common MIC value was ≤ 0.75 μg/mL, accounting for 80.0% of the cases. In addition, the MIC value of 1.0 μg/mL was the second most common at 14.7%, while other categories contributed less than 5%. Although the MIC value of ≤ 0.75 μg/mL increased slightly to 83.3% in 2021 and the proportions of other categories decreased marginally, these changes did not reach statistical differences (*p*=0.763). The following year, a significant change of MIC distribution was observed (*p* < 0.0001). The MIC value of 1.0 μg/mL became the most prevalent at 51.0%, followed by the MIC value of 1.5 μg/mL at 31.8%. Meanwhile, the MIC value of ≤ 0.75 μg/mL showed a significant decline of 69.2%. In 2023, the MIC value of 2.0 μg/mL increased dramatically from 2.5% to 12.2%, while the percentages of MIC values between 1.0 and 1.5 μg/mL declined steadily. In addition, the MIC value of ≤ 0.75 μg/mL experienced a slight increase of 1.3% (*p*=0.001). In 2024, the MIC value of 1.0 μg/mL became the most prevalent, rising to 76.7%. The percentages of MIC values between 1.5 and 2.0 μg/mL dropped significantly to below 10% for each category. Simultaneously, the MIC value of ≤ 0.75 μg/mL decreased slightly to 13.6% (*p* < 0.0001) (Figure 3 and Table 3).

**Figure 4:**
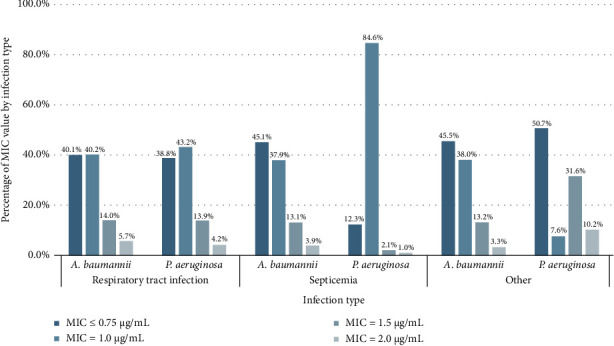
Comparison between colistin MIC value and infection type of *A. baumannii* and *P. aeruginosa*.

Referring to Figure 4, the colistin MIC value of *A. baumannii* and *P. aeruginosa* across different types of infections showed the differences in septicemia and other infections cases. In septicemia cases, the percentage of MIC value at 1.0 μg/mL for *P. aeruginosa* was noticeably higher than that for *A. baumannii*, while the other MIC values for *P. aeruginosa* were significantly lower than those for *A. baumannii* (*p* < 0.0001). Regarding other infections, MIC values for *P. aeruginosa* were predominantly ≤ 0.75 μg/mL and 1.5 μg/mL, accounting for 50.7% and 31.6%, respectively, which were significantly higher than the corresponding values for *A. baumannii* (*p* < 0.0001). In contrast, the MIC distribution in respiratory tract infection cases showed no significant differences (*p*=0.434).

### 3.4. Carbapenem Resistance Characteristics Based on Colistin MIC Values

There was a statistically significant difference in MIC value between carbapenem-resistant strains and nonresistant strains (Table 4). The carbapenem-resistant *A. baumannii* strains' colistin MIC values showed significant differences for the antibiotics meropenem and imipenem (*p* < 0.0001). Specifically, the percentages of MIC values ranging from 1.0 to 2.0 μg/mL were higher in resistant strains compared with nonresistant strains. Carbapenem-resistant *P. aeruginosa* strains did not show statistical differences in colistin MIC values for meropenem, whereas a significant variation was observed for imipenem (*p*=0.031). The percentages of MIC values ranging from 1.0 to 1.5 μg/mL in imipenem-resistant *P. aeruginosa* strains were slightly higher than in nonresistant strains.

## 4. Discussion

Antibiotic resistance, especially in Gram-negative bacteria such as Enterobacteriaceae, *A. baumannii*, and *P. aeruginosa*, poses a global health threat [11]. Colistin serves as a last-resort antibiotic against these strains, yet its efficacy is challenged by emerging drug-resistant strains, notably in *A. baumannii* and *P. aeruginosa* [12]. In this study, there was an increase in *A. baumannii* infections from 2020 to 2024, particularly during the peak of the COVID-19 pandemic in Vietnam, possibly due to factors such as prolonged hospitalizations and increased ventilator use. *A. baumannii* exhibited higher resistance rates to tested antibiotics compared with *P. aeruginosa*, particularly against fluoroquinolones and β-lactams. However, both bacterial species showed no resistance to colistin during the study period.

The incidence of *A. baumannii* and *P. aeruginosa* infections is increasing each year. According to a systematic review by Usman Abubakar, during the COVID-19 pandemic, there was an increase in the incidence of carbapenem-resistant strains of *A. baumannii* and a decrease in *P. aeruginosa* [13]. These findings were consistent with results from the present study, which showed an increase in *A. baumannii* infections from 2020 to 2024, corresponding to the peak of the COVID-19 pandemic in Vietnam. This increase in *A. baumannii* infections was associated with prolonged hospitalizations, increased demand for ventilators, and the use of immunosuppressive drugs, particularly steroids [13]. These conditions could induce a favorable environment for the transmission of *A. baumannii* and *P. aeruginosa*, as they can persist on the surfaces of medical devices, especially ventilators.

The lower respiratory tract specimen, including sputum, bronchial wash, and endotracheal aspirate, is where *A. baumannii* and *P. aeruginosa* are most frequently isolated in the present study. *A. baumannii* and *P. aeruginosa* are recognized as causative agents of hospital-acquired pneumonia and ventilator-associated pneumonia, with high multidrug resistance to commonly used antibiotics [14, 15]. Patients with prolonged hospital stays and immunodeficiency, especially intensive care treatment, are at high risk of *A. baumannii* and *P. aeruginosa* infections. In addition, pus and urine also have a high positive rate for the two bacteria under consideration. These are specimens from primary infection sites where bacteria can penetrate deeper, leading to bloodstream infections and organ involvement [16].

Although the colistin-resistant strains for both *A. baumannii* and *P. aeruginosa* were not observed in the present study, the MIC values of colistin for these bacteria changed. Before 2010, a study by Mezzatesta et al. pointed out that over 90% of the colistin MIC distribution for *A. baumannii* concentrated at ≤ 0.75 μg/mL [17], whereas a study by Tada et al. demonstrated that the Colistin MIC90 for *P. aeruginosa* was 0.5 μg/mL in this period [18]. From 2010 to 2020, the colistin MIC value was higher than the values reported in earlier studies. A study by Al-Sweih, Al-Hubail, and Rotimi reported that the MIC50 and MIC90 of Colistin for *A. baumannii* were 1 μg/mL and 3 μg/mL, respectively, but no resistant strains were observed [19]. Likewise, the most prevalent MIC value of *P. aeruginosa* was reported at 2 μg/mL and only 2% of resistant strains were detected [20–22]. From 2020 to present, a study by Kon et al. conducted on colistin-susceptible *A. baumannii* strains demonstrated that the most prevalent MIC value was 1 μg/mL, accounting for 66.5% [23]. In addition, a study by Nguyen et al. on *P. aeruginosa* strains from a general hospital in Ho Chi Minh City, Vietnam, showed that the majority of strains had MIC values for colistin primarily concentrated at ≤ 0.75 μg/mL, with only 0.8% of strains resistant to colistin [24]. Similarly, a study by Sacco et al. on *A. baumannii* strains from an ICU department of a general hospital in Italy also predominantly exhibited MIC values for colistin at ≤ 0.75 μg/mL [25]. These findings demonstrated that the use of colistin for treating infections caused by *A. baumannii* and *P. aeruginosa* in hospitals still held the potential for effective outcomes [25]. While the MIC values for both bacteria were not resistance, the present study shows that they were approaching the cutoff point of 2 μg/mL, perhaps raising concerns about the potential development of resistance to colistin. This serves as a warning that these bacteria may be rapidly developing resistance to colistin.

*A. baumannii* and *P. aeruginosa* have shown decreased susceptibility to colistin, as evidenced by a progressive increase in colistin MIC values from 2020 to 2024. This may indicate the bacteria becoming less susceptibility to colistin, reflected by a shift in MIC values, although these values are not yet classified as resistant. Resistance in *A. baumannii* and *P. aeruginosa* to colistin primarily occurs through mechanisms such as alterations in outer membrane porins, reduced negative charge on phospholipid structures, or increased expression of efflux pumps through known resistance genes [8]. However, in clinical practice in Vietnam, the detection of colistin-resistance genes in bacteria is not routinely performed as part of the diagnostic process. Therefore, the mechanisms behind the increased MIC values of colistin in *A. baumannii* and *P. aeruginosa* in this study remain unclear. Future studies on the resistance mechanisms of these bacterial species are needed to develop management strategies and promote rational antibiotic use to prevent the emergence of colistin-resistant strains in the future.

Attention to the carbapenem resistance characteristics of colistin-resistant bacterial strains is crucial for safeguarding public health and addressing the growing threat of antibiotic resistance. Antibiotic resistance genes can be transferred between bacteria through plasmids or other mobile genetic elements. Colistin-resistant bacteria could carry carbapenem resistance genes and transfer them to other bacteria, especially in Gram-negative bacteria [26]. In addition, the infections caused by bacteria resistant to both colistin and carbapenem have a significantly higher mortality rates compared with those caused by carbapenem-susceptible bacteria [27]. In Vietnam, colistin is regarded as one of the last-resort antibiotics for treating carbapenem-resistant *A. baumannii* and *P. aeruginosa*. Therefore, clinicians need to assess its in vitro efficacy against these strains to make informed decisions regarding its use. The present study reveals that imipenem-resistant *P. aeruginosa* strains demonstrated higher percentages of colistin MIC values at 1.0 μg/mL and 1.5 μg/mL compared with nonresistant strains. Similarly, *A. baumannii* showed an increased proportion of MIC values ranging from 1.0 to 2.0 μg/mL. This finding is particularly concerning, as it indicates a trend toward rising resistance, which could complicate treatment options for infections caused by these pathogens. Although there is no direct evidence linking colistin use in carbapenem-resistant strains to increase colistin MIC values, careful monitoring of MIC trends is essential to detect the potential emergence of strains resistant to both colistin and carbapenem in the future. Vigilant surveillance of these MIC values in affected strains is vital to detect any shifts in resistance patterns early, allowing for timely interventions to reduce the risk of further resistance development. If no action is taken promptly, the use of antibiotics to treat these bacterial strains will become increasingly limited due to the lack of effective antibiotics available for selection. Therefore, preserving the effectiveness of current antibiotics is crucial until new treatment therapies are discovered for these strains.

The study's findings have important clinical implications. By identifying the statistically significant change in MIC values for colistin among *A. baumannii* and *P. aeruginosa* from 2020 to 2024, the study alerts healthcare providers to the potential development of resistance to colistin in these bacterial strains. This information is crucial for guiding antibiotic treatment decisions, especially in cases of multidrug-resistant infections where colistin is considered one of the last-resort antibiotics.

This study has some major strengths, demonstrating methodological rigor, ethical considerations, and providing valuable insights into antibiotic resistance trends. It makes a significant contribution to the field of antimicrobial resistance research in a Vietnamese context.

Although the results of the present study are promising, there are some methodological limitations to bear in mind that should be addressed in future studies. First, this study was conducted retrospectively on antibiotic resistance data from patient samples collected at the hospital. Consequently, clinical information, such as the source of infection, length of hospital stays, and response to antibiotic treatment, was not gathered. Furthermore, the correlation between antibiogram and patient treatment outcome was rarely investigated. During the implementation of this study, the final outcomes of antibiotic treatment in patients were influenced by various factors, including the patient's underlying health conditions, the quality of antibiotics used, and the patient's economic circumstances, among others. To evaluate the response to antibiotic treatment in patients, a separate study with prospective sampling and close monitoring of each patient's treatment process is required. Finally, a tool for distinguishing between bacterial strains of the same species in repeated samples has not been approached. Next, due to limited resources, the study did not incorporate molecular biology techniques, such as Multi-Locus Sequence Typing (MLST) to identify the Sequence Typing (ST) of these bacteria. That could be helpful to investigate the precise strain or track the evolutionary lineage of these bacterial isolates with high accuracy.

Although traditional identification techniques, such as Gram staining and biochemical reactions, may be considered less precise compared to molecular techniques, they are still commonly used due to their cost-effectiveness and ability to provide relatively reliable identification in resource-limited hospital settings like ours [28]. Despite being a gold standard method for determining the MIC of antibiotics, the broth microdilution (BMD) method is not routinely performed for diagnostic testing in most Vietnamese healthcare facilities due to its high cost and more complex technical requirements. Some hospitals that utilize automated antibiogram systems only apply the Colistin broth disk elution (CBDE) method, which is accepted by the CLSI as an alternative to BMD in clinical practice settings. However, as of 2024, the CLSI has only approved CBDE for Enterobacterales and *P. aeruginosa*. Prior to September 2024, our laboratory had not yet implemented the CBDE method. In the present study, the E-test method was chosen for determining colistin MIC values because it is a routine technique commonly used in diagnostic laboratories in Vietnam. This method is faster, less time-consuming, and more accessible as a standard procedure. Moreover, the E-test provides detailed MIC values, which are instrumental in accurately identifying heteroresistant subpopulations [23]. Anticipated outcomes from this study are expected to contribute valuable data for future research addressing these dangerous strains. These research results come from real-world data in diagnosing pathogens and describing local antibiotic resistance patterns. While more advanced methods were not employed to improve test accuracy, the data presented here hold significant value, closely reflecting real-world information accessible to healthcare professionals regarding bacterial patterns and colistin MIC trends in clinical practice. Our results provide evidence of colistin resistance development in bacteria. This implies that colistin MIC monitoring needs to be supervised closely for early detection of resistance. In addition, the data provide a valuable overview of the burden of infections caused by these pathogens, both in Vietnam as a whole and specifically at our hospital.

In future longitudinal studies, monitoring evolving trends in antibiotic resistance, including changes in MIC values over time, will provide insights into the dynamics of resistance development. In addition, assessing clinical outcomes of patients treated with colistin, considering factors such as treatment response, adverse effects, and long-term outcomes, is crucial. Furthermore, employing molecular biology techniques, such as the 16S rDNA sequencing to determine bacterial species, along with genomic analysis techniques to identify genetic determinants of antibiotic resistance in *A. baumannii* and *P. aeruginosa* strains, will contribute to understanding the spread and evolution of resistance genes. Due to the incomplete development of localized antibiotic resistance datasets for bacteria in hospitals across Vietnam, the data remain fragmented and lacks regular updates across different hospitals. This issue will be addressed in the future once these datasets are fully established.

## 5. Conclusion

From 2020 to 2024, despite no colistin-resistant strains for *A. baumannii* and *P. aeruginosa* being detected, the results demonstrate a statistically significant change in the most prevalent colistin MIC values from ≤ 0.75 μg/mL to 1.0 μg/mL by approximately 60% for two bacteria of interest. Further advanced studies are needed to investigate the changes in colistin MIC values, understand the resistance mechanisms of these bacteria, and develop appropriate strategies for antibiotic management and usage before the resistance becomes complete.

## Figures and Tables

**Table 1:** The percentage of *A. baumannii* and *P. aeruginosa* from 01/2020 to 12/2024 and the percentage of monoinfection and coinfection per year.

Bacteria	2020		2021		2022		2023		2024		Total of 5 years	
	*N*	%	*N*	%	*N*	%	*N*	%	*N*	%	*N*	%
*A. baumannii*	305	11.1	371	16.0	532	18.1	694	20.3	578	20.5	2480	17.4
Monoinfection caused only by *A. baumannii*	196	64.3	271	73.0	347	65.2	536	77.2	433	74.9	1783	71.9
Coinfection caused by *A. baumannii* and other bacteria	109	35.7	100	27.0	185	34.8	158	22.8	145	25.1	697	28.1

*P. aeruginosa*	268	9.7	216	9.3	289	9.8	343	10.0	249	8.8	1365	9.6
Monoinfection caused only by *P. aeruginosa*	180	67.2	138	63.9	209	72.3	250	72.9	182	73.1	959	70.3
Coinfection caused by *P. aeruginosa* and other bacteria	88	32.8	78	36.1	80	27.7	93	27.1	67	26.9	406	29.7

Common multidrug-resistant Gram-negative bacteria (⁣^∗^)	2755	100.0	2315	100.0	2941	100.0	3427	100.0	2814	100.0	14,252	100.0

⁣^∗^Common multidrug-resistant gram-negative bacteria consists of *A. baumannii*, *P. aeruginosa*, *K. pneumoniae*, *E. coli* and *Enterobacter* spp.

**Table 2:** The resistance rates to the tested antibiotics of *A. baumannii* and *P. aeruginosa*.

Tested antibiotics	*A. baumannii*			*P. aeruginosa*		
	S *n* (%)	I *n* (%)	R *n* (%)	S *n* (%)	I *n* (%)	R *n* (%)
Piperacillin/tazobactam	252 (13.2%)	72 (3.8%)	1576 (83.0%)	660 (73.2%)	127 (14.1%)	115 (12.8%)
Amoxicillin/clavulanate	No tested			8 (2.6%)	0 (0.0%)	296 (97.37%)
Ticarcillin/clavulanate	No tested			499 (55.3%)	139 (15.4%)	264 (29.3%)
Trimethoprim/sulfamethoxazole	501 (26.1%)	62 (3.2%)	1357 (70.7%)	22 (2.6%)	45 (5.3%)	780 (92.1%)
Doxycycline	853 (46.8%)	178 (9.8%)	792 (43.4%)	No tested		
Amikacin	No tested			717 (76.0%)	14 (1.5%)	212 (22.5%)
Gentamicin	378 (19.2%)	25 (1.3%)	1561 (79.5%)	631 (68.7%)	28 (3.1%)	259 (28.2%)
Tobramycin	451 (23.4%)	43 (2.2%)	1437 (74.4%)	651 (72.3%)	5 (0.6%)	244 (27.1%)
Netilmicin	No tested			711 (75.9%)	5 (0.5%)	221 (23.6%)
Ciprofloxacin	215 (10.7%)	36 (1.8%)	1765 (87.5%)	555 (59.1%)	47 (5.0%)	337 (35.9%)
Levofloxacin	254 (13.0%)	38 (1.9%)	1659 (85.1%)	496 (54.5%)	80 (8.8%)	335 (36.7%)
Cefotaxime	48 (2.4%)	247 (12.3%)	1718 (85.3%)	No tested		
Ceftazidime	280 (13.9%)	37 (1.8%)	1704 (84.3%)	676 (71.7%)	17 (1.8%)	250 (26.5%)
Cefepime	310 (15.5%)	40 (2.0%)	1645 (82.5%)	676 (72.4%)	8 (0.9%)	249 (26.7%)
Imipenem	303 (15.1%)	20 (1.0%)	1688 (83.9%)	671 (71.5%)	27 (2.9%)	241 (25.7%)
Meropenem	261 (14.2%)	4 (0.2%)	1575 (85.6%)	538 (62.4%)	32 (3.8%)	278 (32.8%)
Colistin	0.0%	2027 100.0%	0.0%	0.0%	946 100.0%	0.0%

*Note:* No.

**Table 3:** The MIC value ranges of *A. baumannii* and *P. aeruginosa*.

	MIC ≤ 0.75 μg/mL *N* (%)	MIC = 1.0 μg/mL *N* (%)	MIC = 1.5 μg/mL *N* (%)	MIC = 2.0 μg/mL *N* (%)	*p* value⁣^∗^
*A. baumannii*					
2020	330 (82.9%)	38 (9.5%)	20 (5.0%)	10 (2.5%)	0.909^a^ < 0.0001^b^ 0.004^c^ < 0.0001^d^
2021	222 (84.4%)	25 (9.5%)	11 (4.2%)	5 (1.9%)	
2022	95 (24.0%)	186 (47.0%)	97 (24.5%)	18 (4.5%)	
2023	99 (18.2%)	254 (46.8%)	134 (24.7%)	56 (10.3%)	
2024	92 (21.5%)	301 (70.5%)	18 (4.2%)	16 (3.7%)	
Total	838 (41.3%)	804 (39.7%)	280 (13.8%)	105 (5.2%)	

*P. aeruginosa*					
2020	136 (80.0%)	25 (14.7%)	5 (2.9%)	4 (2.4%)	0.763^a^ < 0.0001^b^ 0.001^c^ < 0.0001^d^
2021	110 (83.3%)	18 (13.6%)	3 (2.3%)	1 (0.8%)	
2022	29 (14.6%)	101 (51.0%)	63 (31.8%)	5 (2.5%)	
2023	43 (15.9%)	130 (48.1%)	64 (23.7%)	33 (12.2%)	
2024	24 (13.6%)	135 (76.7%)	13 (7.4%)	4 (2.3%)	
Total	342 (36.2%)	409 (43.2%)	148 (15.6%)	47 (5.0%)	

⁣^∗^Chi-square and Fisher's exact test.

^a^The difference between MIC value in 2020 and MIC value in 2021.

^b^The difference between MIC value in 2021 and MIC value in 2022.

^c^The difference between MIC value in 2022 and MIC value in 2023.

^d^The difference between MIC value in 2023 and MIC value in 2024.

**Table 4:** Carbapenem resistance characteristics based on colistin MIC values of *A. baumannii* and *P. aeruginosa*.

Carbapenem resistance		Colistin MIC values				Total *N* (%)	*p* value⁣^∗^
		MIC ≤ 0.75 μg/mL *N* (%)	MIC = 1.0 μg/mL *N* (%)	MIC = 1.5 μg/mL *N* (%)	MIC = 2.0 μg/mL *N* (%)		
*A. baumannii*							
Imipenem	Resistance	606 (35.9%)	736 (43.6%)	250 (14.8%)	96 (5.7%)	1688 (100%)	< 0.0001
	No resistance	219 (67.8%)	66 (20.4%)	29 (9.0%)	9 (2.8%)	323 (100%)	
Meropenem	Resistance	569 (36.1%)	689 (43.7%)	226 (14.3%)	91 (5.8%)	1575 (100%)	< 0.0001
	No resistance	184 (69.4%)	51 (19.2%)	24 (9.1%)	6 (2.3%)	265 (100%)	

*P. aeruginosa*							
Imipenem	Resistance	70 (29.1%)	122 (50.6%)	39 (16.2%)	10 (4.1%)	241 (100%)	0.031
	No resistance	268 (38.4%)	285 (40.8%)	108 (15.5%)	37 (5.3%)	698 (100%)	
Meropenem	Resistance	92 (33.1%)	133 (47.8%)	37 (13.3%)	16 (5.8%)	278 (100%)	0.301
	No resistance	214 (37.5%)	236 (41.4%)	90 (15.8%)	30 (5.3%)	570 (100%)	

⁣^∗^Chi-square test.

## Data Availability

The data that support the findings of this study are available from the corresponding author upon reasonable request.
